# Children's Physical Self-Concept, Motivation, and Physical Performance: Does Physical Self-Concept or Motivation Play a Mediating Role?

**DOI:** 10.3389/fpsyg.2021.669936

**Published:** 2021-04-30

**Authors:** Annette Lohbeck, Philipp von Keitz, Andreas Hohmann, Monika Daseking

**Affiliations:** ^1^Educational Sciences, University of Paderborn, Paderborn, Germany; ^2^Educational Psychology, Helmut Schmidt University, Hamburg, Germany; ^3^Cultural Studies, Sport Science I, University of Bayreuth, Bayreuth, Germany

**Keywords:** physical self-concept, intrinsic motivation, extrinsic motivation, physical performance, relations

## Abstract

The present study aimed to examine the relations between physical self-concept, intrinsic and extrinsic motivation as well as physical performance of 1,082 children aged 7–8 years. The central objective of this study was to contrast a mediation model assuming physical self-concept as a mediator of the relations between both types of motivation and physical performance to a mediation model assuming both types of motivation as mediators of the relations between physical self-concept and physical performance. Physical self-concept and both types of motivation were measured by using self-reported questionnaires, while physical performance was measured with 10 motor skill tests. All tests were carried out during regular school hours (8–12 A.M.) by qualified test personnel. Beyond correlation analyses, structural equation modeling (SEM) was performed to find evidence for the predictive relations between the variables under study. Results showed that physical self-concept was significantly positively related to both types of motivation and physical performance (all *p* < 0.001). In contrast, results of SEM revealed that only physical self-concept (*p* < 0.001) and intrinsic motivation (*p* < 0.05) were significantly positively linked to physical performance. Furthermore, physical self-concept proved to significantly mediate the relations of both types of motivation to physical performance (*p* < 0.001), while only intrinsic motivation, but not extrinsic motivation, proved to significantly mediate the relation between physical self-concept and physical performance (*p* < 0.05). These results suggest that school-based or extracurricular interventions targeted at improving younger children's physical performance only by means of an increased level of physical activity or by external factors without supporting children's physical self-concept and intrinsic motivation may have less or no effects on their physical performance.

## Introduction

**Figure 1:**
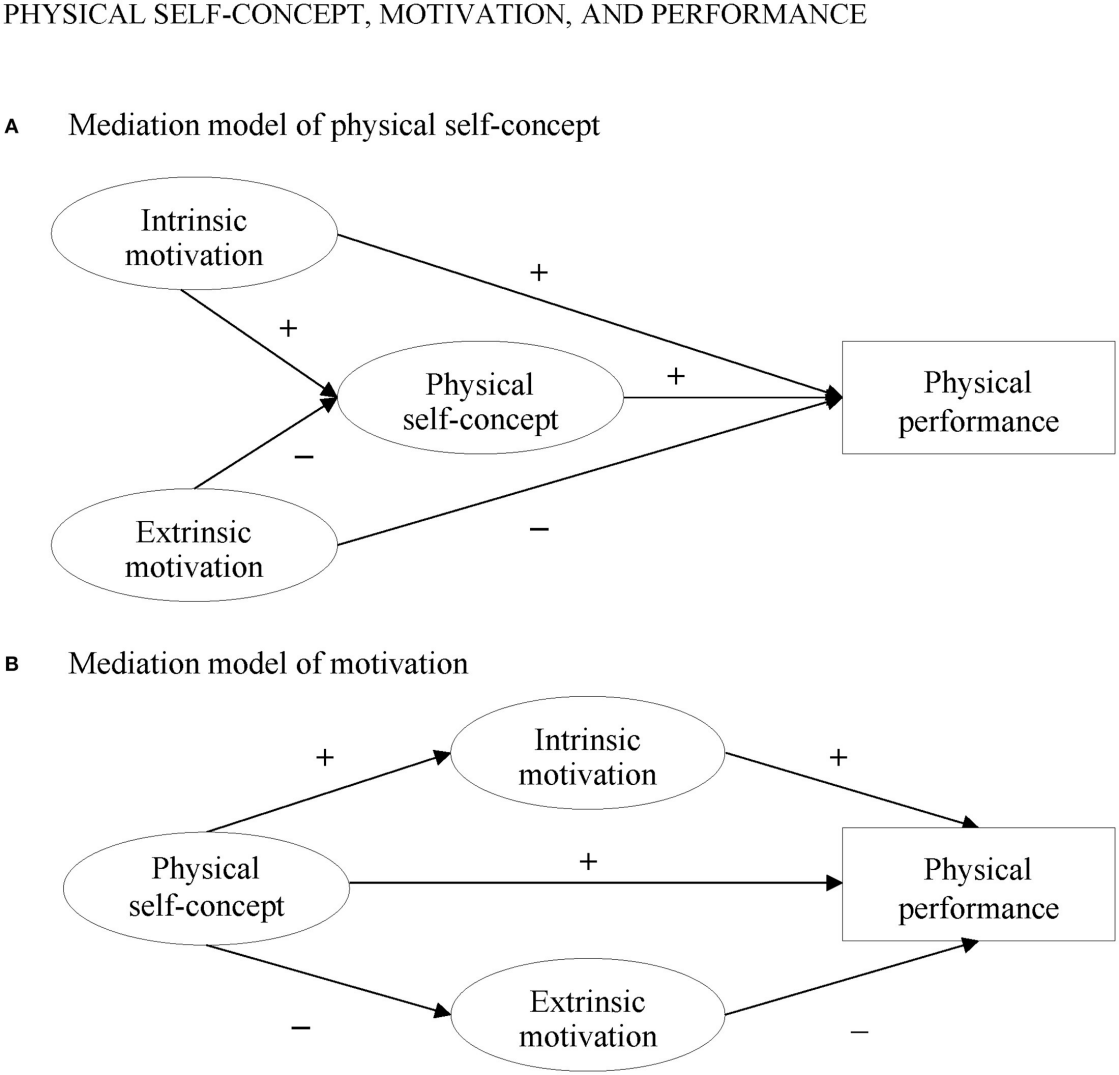
Mediation models under investigation. **(A)** Mediation model of physical self-concept. **(B)** Mediation model of motivation.

Physical activity has profound positive effects on health and well-being of young children (Eime et al., 2013; Richter et al., 2016; Brown et al., 2017; Rodriguez-Ayllon et al., 2019). However, of great concern are the increasing decline of children's participation in physical activities (Eime et al., 2016) and the high prevalence of obesity in childhood (Bodzsar and Zsakai, 2014). Over 340 million children and adolescents aged 5–19 were obese or overweight in 2016 (World Health Organization, 2016). Research on the underlying motivational processes that account for varying levels of younger children's physical activity is thus of considerable importance for health enhancement and the promotion of children's physical performance, especially in early childhood when children's physical abilities increasingly develop (Lakes et al., 2020; Schmutz et al., 2020). For this reason, the present study aimed to examine the possible determinants of younger children's physical performance at the early start of their development. More precisely, this study strived to explore the relations between physical self-concept, intrinsic and extrinsic motivation as well as physical performance of children aged 7–8 years. The central objective of this study was to contrast two mediation models to each other within the structural equation modeling (SEM) framework to provide deeper insight into the interplay of physical self-concept, motivation, and physical performance: The first mediation model assumes that physical self-concept is a mediator for the relations between both types of motivation and physical performance, that is children need to be motivated in physical activities to develop a more positive self-concept that, in turn, improves physical performance. In contrast, the second mediation model posits that both types of motivation are mediators of the relations between physical self-concept and physical performance, that is children need to perceive themselves as competent in physical activities (i.e., show a positive physical self-concept) to increase or maintain their motivation, leading to better physical performance. Both mediation models thus differ in terms of their predictive power on adherence to regular physical exercise in the pediatric population: While the first model proposes that a high level of motivation is essential to develop a positive physical self-concept and to perform well in physical activities, the second mediation model suggests that it is rather a high physical self-concept that is needed to increase motivation and to improve physical performance. However, to the best of our knowledge, no research has tested both models in one empirical study simultaneously. As a consequence, it is still unclear in the literature whether the first model assuming physical self-concept as a mediator would be more rigorous than the mediation model assuming both types of motivation as mediators. To find answers to this research question, both mediation models were tested simultaneously in this study. Figure 1 depicts both mediation models under investigation.

### Physical Self-Concept and Motivation

Physical self-concept and motivation are two important factors that influence children's physical performance. Physical self-concept represents a specific domain within the hierarchical self-concept model developed by Shavelson et al. (1976) and describes how individuals perceive their abilities in physical domains (Marsh and Redmayne, 1994; Marsh, 2007). Due to its great impact on various educational outcomes, physical self-concept has caught considerable interest in research of sport sciences and psychology (Mendo-Lázaro et al., 2017; Fernández-Bustos et al., 2019; Onetti-Onetti et al., 2019). For instance, many studies have provided evidence for a reciprocal effects model between physical self-concept and physical achievement, that is prior physical self-concept/achievement predicts subsequent physical achievement/self-concept that, in turn, predicts subsequent physical self-concept/achievement (i.e., Marsh et al., 2006a,b, 2007a; Trautwein et al., 2008; Garn et al., 2019). Beyond a positive self-concept, motivation has also been found as a strong predictor of physical achievement [see Ntoumanis and Standage (2009), Cerasoli et al. (2014) for a meta-analysis]. Motivation describes the internal and/or external forces that produce the initiation, direction, intensity, and persistence of a behavior (cf., Vallerand, 2007, p. 59). In the present study, we focused on the dichotomy of motivation, that is intrinsic and extrinsic motivation. Intrinsic motivation is characterized by a completely internal regulation of a behavior, referring to all behaviors that are performed for the inherent pleasure emanating from an activity (Deci and Ryan, 2002). In contrast, extrinsic motivation is defined as the external control of a behavior, referring to all behaviors that are governed by the consequences of an activity or externally controlled constraints such as rewards or threats (Vasconcellos et al., 2020). Numerous studies have shown that these two types of motivation were differentially related to physical achievement (e.g., Craggs et al., 2011; Standage et al., 2012; Wang et al., 2016). While intrinsic motivation has been found to positively relate to physical achievement (e.g., Standage et al., 2003, 2008; Gillet et al., 2010), extrinsic motivation has been found to negatively relate to physical achievement (e.g., Vallerand, 2007; Boiché et al., 2008; Gillet et al., 2010).

### Relations Between Physical Self-Concept, Motivation, and Physical Performance

A central theoretical rationale for proposing relations between physical self-concept, motivation, and physical achievement is expectancy–value theory (Eccles and Wigfield, 2020). This theory assumes that expectancies of success reflect individuals' ability beliefs (i.e., self-concepts) that influence many achievement-related outcomes such as achievement. For this reason, we hypothesize that children who feel competent and are more motivated in physical activities perform better in physical activities than children with a more negative physical self-concept and lower motivation. Physical self-concept and motivation should therefore predict physical performance. However, most studies with younger children have investigated the relations between self-concept and achievement in academic domains (e.g., Nicholls, 1979; Stipek and MacIver, 1989; Wigfield, 1994; Marsh et al., 2002; Arens et al., 2016). In contrast, very little research has yet explored the relations between physical self-concept, motivation, and physical achievement of younger children in the physical domain, especially in early childhood. As a consequence, it is still unknown whether physical self-concept or motivation plays a mediating role in predicting physical performance of younger children at earlier developmental stages. For this reason, the present study sought to assess the possible mediating effects of physical self-concept and the two types of motivation (i.e., intrinsic and extrinsic motivation) on younger children's physical performance in early childhood. Some evidence for the mediating role of physical self-concept (e.g., Ntoumanis, 2001; Standage et al., 2003; Thøgersen-Ntoumani and Ntoumanis, 2006; Sproule et al., 2007; Cumming et al., 2011) or the mediating role of motivation have already been found in previous studies (e.g., Marsh et al., 2005; McDonough and Crocker, 2007; Guay et al., 2008). However, those studies have not tested the mediating role of physical self-concept or motivation in younger samples of children at the early start of their development.

### Gender and Physical Differences

In early childhood, children typically show a very positive physical self-concept and high motivation in physical activities, which increasingly decline during adolescence (Marsh et al., 1998; Harter, 2012). Although gender differences may be less prevalent in younger children before adolescence, boys usually show a more positive physical self-concept and higher motivation as well as better physical performance than girls (e.g., Eccles and Harold, 1991; Jacobs et al., 2002; Morano et al., 2011). In addition, several studies have revealed that children's body mass index (BMI), calculated as weight (kg) divided by height (m) squared, was closely intertwined with differences in physical self-concept (Paeratakul et al., 2002; Marsh et al., 2007b; Morano et al., 2011), motivation, and physical achievement (Trost et al., 2001; Morano et al., 2011; Zsakai et al., 2017): overweight or obese children typically demonstrate a lower physical self-concept, lower motivation, and poorer performance in physical activities than their normal-weight peers. However, it is still unclear in the literature whether children's BMI also changes the relations between physical self- concept, motivation, and physical performance (i.e., does being obese reinforce or decrease the relations?). For this reason, it is important to control for children's sex and BMI when exploring the relations between children's physical self-concept, motivation, and physical performance.

## Objectives and Hypotheses

Drawing on a very neglected sample of children aged 7–8 years, the present study aimed to examine the relations between physical self-concept, intrinsic and extrinsic motivation as well as physical performance. One of the central objectives of this study was, in particular, that two mediation models were contrasted to each other: a mediation model assuming physical self-concept as a mediator of the relations between both types of motivation and physical performance to a mediation model assuming both types of motivation as mediators of the relations between physical self-concept and physical performance. Despite the rather exploratory nature of this study, the theoretical and empirical framework allowed us to suggest the following three hypotheses.

Hypothesis 1: Physical self-concept is positively related to intrinsic motivation and physical performance but negatively related to extrinsic motivation.
Hypothesis 2: Both physical self-concept and motivation will predict physical performance, that is a more positive physical self-concept and higher levels of intrinsic motivation will positively predict physical performance, while higher levels of extrinsic motivation will negatively predict physical performance.
Hypothesis 3: There is support for both a mediation model of physical self-concept and a mediation model of motivation.

## Method

### Participants

The data set used in this study is part of the larger Fulda Movement Check project that was first introduced as a campaign in Fulda for sustainable movement and health, sport, and talent promotion in 2010. Since then, this project has been pursued as a regular annual and comprehensive sports motor assessment (screening) in the second grade of elementary schools in Fulda. With a number of *N* > 13,500 children so far, the Fulda Movement Check project provides meaningful data sets and findings on the performance of younger children aged 7–8 years in Fulda (Hohmann et al., 2016). The sample of this study was drawn from this project and consisted of 1,082 children aged 7–8 years from 64 schools in Fulda. Of these, *n* = 526 children were boys (48.6 %), and *n* = 556 children were girls (51.4 %). Most of the participating children (68.1%) were 7 years old (*M* = 7.32; SD = 0.47).

### Procedure

The Fulda Movement Check was performed in different sports halls in Fulda during 4 weeks with different school classes. All participants were assessed under similar conditions. The tests were carried out during regular school hours (8–12 A.M.) by qualified test personnel. For the present study, the children were divided into two groups because the entire hall was needed for the 6-min endurance run tested in this project. While one group performed the endurance run, the other group filled out the questionnaires of physical self-concept and both types of motivation. All items were read aloud by a trained student assistant to make sure that all children were able to adequately respond to the items of the questionnaires. Before the responding to the measures began, it was clearly emphasized to the children that there were no right or wrong responses and that they could withdraw from the study at any time without any negative consequences. The responding of the questionnaire took approximately 10–15 min. Participation was voluntary and anonymous. Before entering the test campaign, all children's parents provided written informed consent for the recording and scientific use of the data collected in this study. The district head administration office of Fulda supervised this project in cooperation with the University of Bayreuth, the state education authority of Fulda, the sports department of Fulda, the sports area of Fulda, the participating schools, and the sport clubs of Fulda.

### Measures

#### Physical Self-Concept

To measure children's physical self-concept, three slightly modified items of the Self-Description–Questionnaire I (Marsh, 1990) were used. All three items were positively worded and formulated as questions that were deemed more appropriate for younger children than statements (Marsh et al., 2002). The three items were: “Are you good in sports?,” “Are you a good athlete?,” and “Are you athletic?” All three items were followed by a four-point rating scale ranging from 1 = no to 2 = rather no to 3 = rather yes, and to 4 = yes. The reliability of this scale was good (α = 0.80). The selection of these three items was based on previous research using these three items with preschool children (e.g., Marsh et al., 1991, 2002; Arens et al., 2016), in which these items showed the highest factor loadings in confirmatory factor analysis (CFA) and the highest reliability estimates.

### Motivation

Both types of motivation were measured with three slightly modified items from the Academic Self-Regulation Questionnaire (SRQ-A; Ryan and Connell, 1989). These three items have been found as most reliable and valid in previous research as indicated by superior factor loadings and high reliability estimates (Freund and Lohbeck, 2020; Lohbeck et al., 2021^1^). Because the SRQ-A focuses on motivation toward school in general, all items were referred to the physical domain. Like the self-concept scale, all items were formulated as questions, beginning with the stem: “Why do you do sports? Do you do sports because…,” followed by the reasons described in the three items of intrinsic motivation (i.e., “…you like sports?,” “…you are interested in sports?,” and “… you enjoy sports?”) and the three items of extrinsic motivation (i.e., “…you want to be good in competitions?,” “…you want to perform better than other children in competitions?,” and “…you want to have a good result in competitions?”). The reliability of both scales was satisfactory (i.e., α = 0.81 for intrinsic motivation and α = 0.74 for extrinsic motivation).

### Physical Performance

All children participated in the Fulda Movement Check that is based on the German Motor Skills Test 6-18 (in German: Deutscher Motoriktest 6-18; Utesch et al., 2018). For the present study, the following 10 tests were considered as indicators of children's physical performance:

20-m sprint (speed): To measure children's speed, all children performed a 20-m linear running sprint. The starting position was 0.3 m behind the start line, and children had two possible attempts with a break of at least 2 min between the two running sprints. The reliability of this test was *r*_tt_ = 0.90 (Boes and Schlenker, 2016).
Sideward jumping (coordination): To test children's coordination, children were asked to jump sideward with two legs within two adjacent 50 × 50 cm squares without touching a boundary line. They had five trial jumps before the testing and two possible attempts with a break of at least 2 min. This test took 15 s, in which the number of children's sideward jumps was recorded. The objectivity of this test was *r*_obj_ = 0.99, and the reliability was *r*_tt_ = 0.89 (Boes and Schlenker, 2016).
Balancing backwards (coordination): The balancing backwards test was another coordination test in which the children had to balance backwards on three beams (6, 4.5, and 3 cm). For each beam, the number of steps backwards balanced (feet fully raised) until leaving the beam was counted. The maximum number of steps per attempt was limited to eight. For each of the three beams, the children had two possible attempts, resulting in a maximum of 48 steps. The objectivity of this test was *r*_obj_ = 0.99, and the reliability was *r*_tt_ = 0.73 (Utesch et al., 2018).
Standing torso bend forward (flexibility): To measure children's flexibility, children were asked to bend forward as far as possible with their fingertips beyond their feet. They had to hold the best position for at least 3 s and had two possible attempts. The distance of the fingers in centimeters to ground level was recorded, whereby a low range above ground level was recorded as a negative distance. The objectivity of this test was *r*_obj_ = 0.99, and the reliability was *r*_tt_ = 0.94 (Boes and Schlenker, 2016).
Push-ups (strength endurance): The push-ups test targeted at measuring children's strength endurance. In this test, children were requested to touch their hands with each other when the body was lying down on the floor and the arms were extended after the push-up. A complete repetition was evaluated when the upper body was laid down on the mat and the hands touched each other. Children had only one attempt, and the number of correctly executed push-ups within 40 s was recorded. The objectivity of this test was *r*_obj_ = 0.98, and the reliability was *r*_tt_ = 0.69 (Boes and Schlenker, 2016).
Sit-ups (strength endurance): The sit-up test was also carried out to measure children's strength endurance in 40 s. After a short practice phase, only one test was performed, and the number of correctly executed sit-ups was assessed. The objectivity of this test was *r*_obj_ = 0.92, and the reliability was *r*_tt_ = 0.74 (Klein et al., 2012).
Standing long jump (speed): In the standing long jump test, the distance of two-leg standing jumps in centimeters (measured from the heel) was measured. Children had two possible attempts with a break of at least 2 min, but no practice phase was permitted before the testing. The objectivity of this test was *r*_obj_ = 0.99, and the reliability was *r*_tt_ = 0.89 (Boes and Schlenker, 2016).
6-min endurance run (endurance): To measure children's endurance, the number of meters in a 6-min endurance run around a volleyball pitch (9 × 18 m) was recorded. The test was conducted in groups of 15 children at the same time.
Ball throw (strength): The ball throw test was executed with a ball weight of 80 g and with both feet on the ground behind a line. No step or run-up was allowed. The throwing distance was assessed perpendicular to a measuring tape attached to the floor. Accuracy was 0.1 meter. This test showed a high test–retest reliability of *r*_tt_ = 0.82 (*p* < 0.001; *n* = 3,193; Hohmann et al., 2018).
Agility test (coordination and speed): The agility test consisted of 10 runs of 2 m forth and 2 m back into four different directions according to four different colors presented in random order on a computer screen. Each signal was self-triggered by a buzzer. The split-half reliability of the agility test was *r*_tt_ = 0.78 (*p* < 0.001; *n* = 131; Hohmann et al., 2018).

### Physical Characteristics

Beyond the 10 motor skill tests, children's body height and body weight were also measured according to standardized test prescriptions (Hawes and Martin, 2001; Stewart et al., 2011): body height was measured to the nearest 0.1 cm (seca height tester), and body weight was measured to the nearest 0.1 kg (calibrated seca alpha 770).

### Statistical Analyses

All correlational and SEM analyses were performed in M*plus* 8.5 (Muthén and Muthén, 1998–2018) using the robust maximum likelihood estimator and the full information maximum likelihood approach. Missing values were negligible, ranging from 0 to 0.4% on the item level. To evaluate the fit of the models, chi-square test statistics, comparative fit index (CFI), Tucker–Lewis index (TLI), and root mean square error of approximation (RMSEA) with its 90% confidence interval were considered. A good model fit was assumed with CFI/TLI values > 0.90 and RMSEA values < 0.06 (Hu and Bentler, 1999). When testing the two mediation models for this study (see Figure 1), the “model indirect” option implemented in M*plus* was applied. Children's sex and BMI were included as control variables in all models under investigation. All dichotomous variables were *z*-standardized prior to the analyses. For easier interpretation, all *z*-standardized scores of the 10 tests in the Fulda Movement Check were added up to a total performance score and averaged because the results of these 10 tests were based on different scores (e.g., meters, time, and numbers). For this reason, the results of the 20-m sprint, standing torso bend forward, and agility test were recoded such that higher values indicated higher physical performance. Upon request, the authors can send the specific results of the 10 tests obtained in this study.

## Results

### Descriptive Statistics and Correlations

Descriptive statistics and latent (inter-)correlations of all variables are presented in Table 1. As expected, younger children showed a very high physical self-concept and high levels of motivation. Their total performance score ranged from 22.82 to 163.97 (*M* = 116.97, SD = 15.65; modus: 102.64; median: 117.79). All item factor score correlations were significantly positive, ranging from 0.21 to 0.38. Both types of motivation were significantly positively correlated with each other.

**Table 1:** Descriptive statistics and latent (inter-)correlations between the variables under investigation.

**Variables**	**Min**	**Max**	***M (SD)***	**Intrinsic motivation**	**Extrinsic motivation**	**Physical performance**
Physical self-concept	1	4	3.51 (0.76)	0.38^***^ (0.05)	0.38^***^ (0.04)	0.33^***^ (0.04)
Intrinsic motivation	1	4	3.73 (0.64)	–	0.21^***^ (0.04)	0.19^***^ (0.04)
Extrinsic motivation	1	4	3.12 (0.99)		–	0.13^***^ (0.04)
Physical performance	22.82	163.78	116.97 (15.65)			–

*** *p < 0.001*.

### Preliminary Analysis

As a prerequisite of all further analyses, CFA was first performed to test the measurement separability of children's physical self-concept and motivation. More specifically, the following two models were estimated: (1) a two-factor model assuming two distinct factors for physical self-concept and both types of motivation and (2) a three-factor model differentiating between three distinct factors for physical self-concept, intrinsic motivation, and extrinsic motivation. Results of the fit of these models are provided in Table 2.

**Table 2:** Goodness-of-fit statistics and information criteria of the models under investigation.

**Models**	***χ*^2^**	***df***	***CFI***	***TLI***	***RMSEA [90% CI]***
2-factor model	589.132	26	0.695	0.578	0.041 [0.132, 0.152]
3-factor model	36.335	24	0.993	0.990	0.022 [0.000, 0.035]
Predictive model	96.250	43	0.978	0.967	0.034 [0.025, 0.043]
Mediation model of self-concept	73.834	43	0.988	0.981	0.026 [0.015, 0.036]
Mediation model of motivation	96.250	43	0.978	0.967	0.024 [0.025, 0.043]

*χ^2^, chi square; df, degrees of freedom; CFI, comparative fit index; TLI, Tucker-Lewis Index; RMSEA, root mean square error of approximation; CI, confidence interval*.

The three-factor model showed a superior fit to the data when compared to the two-factor model, indicating that children participating in this study were able to clearly differentiate between their physical abilities and both types of motivation.

### Physical Self-Concept and Motivation as Predictors

**Figure 2:**
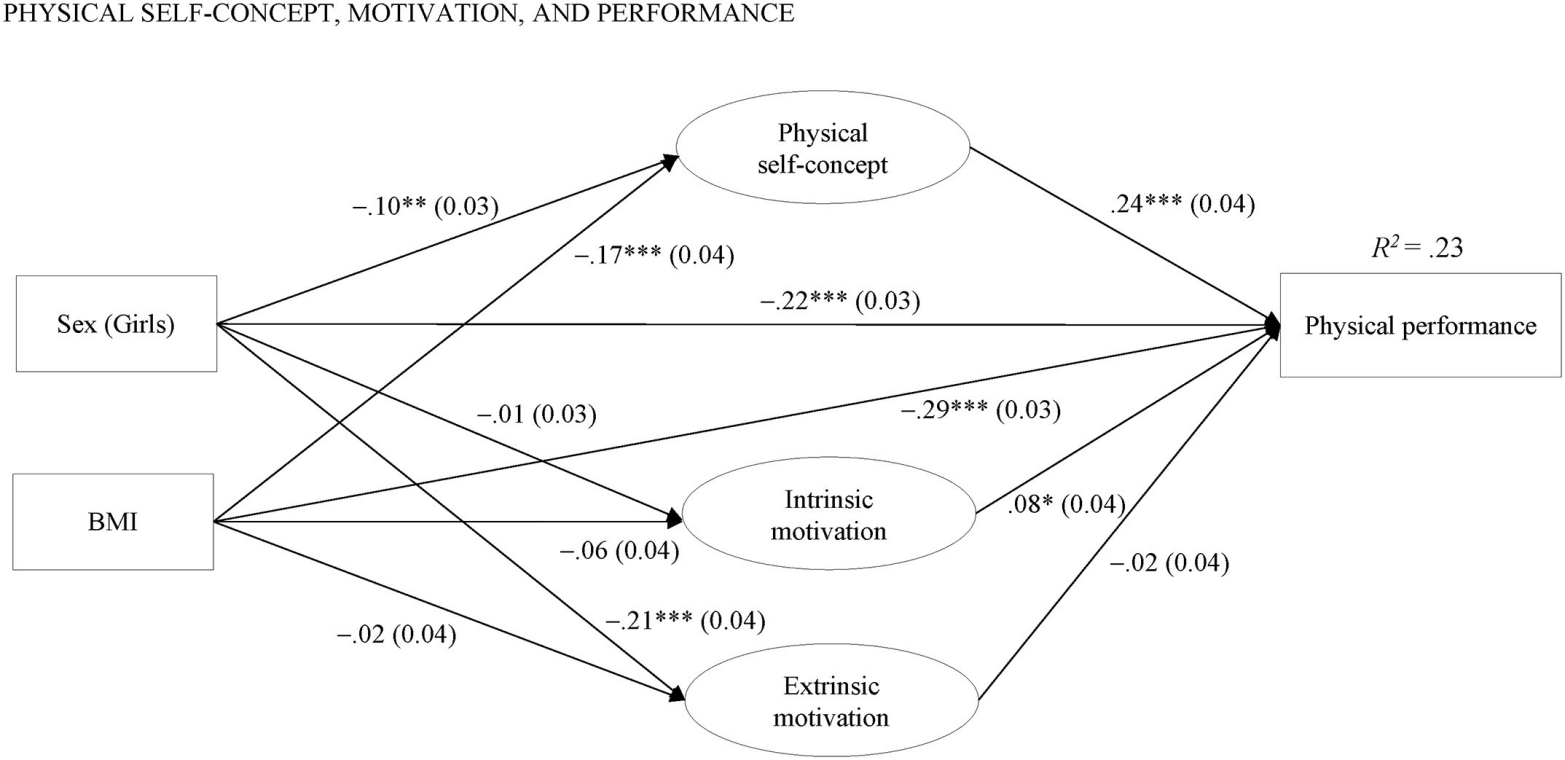
Predictive model under investigation. ***p < 0.001, **p < 0.01, *p < 0.05. *R*^2^ = explained variance.

Results of SEM testing the relations between physical self-concept, both types of motivation (i.e., intrinsic, extrinsic), and physical performance are depicted in Figure 2.

Both physical self-concept and intrinsic motivation were significantly positively related to the total performance score, while extrinsic motivation was slightly negatively but not significantly related to the total performance score. Girls and children with a higher BMI showed a significantly lower total performance score and a lower physical self-concept than boys and children with a lower BMI. Furthermore, girls exhibited significantly higher levels of extrinsic motivation than boys. The fit of this model was good (see Table 2), and the amount of explained variance in the children's total performance score was 0.23.

### Physical Self-Concept and Motivation as Mediators

Results of both mediation models exploring the possible mediating effects of physical self-concept and motivation on physical performance are provided in Table 3.

**Table 3:** Standardized path coefficients (standard errors in brackets) of the mediation models.

	**Mediation model of physical self-concept**				**Mediation model of motivation**			
**Standardized direct effects**	**PSC**	**INTR**	**EXTR**	**PP**	**PSC**	**INTR**	**EXTR**	**PP**
Sex (girls)	−0.02 (0.03)	−0.01 (0.03)	−0.21^***^ (0.04)	−0.22^***^ (0.03)	−0.19^**^ (0.07)	0.05 (0.06)	−0.35^***^ (0.07)	−0.45^***^ (0.06)
BMI	−0.15^***^ (0.04)	−0.06 (0.04)	−0.02 (0.04)	−0.29^***^ (0.03)	−0.07^***^ (0.02)	0.00 (0.02)	0.02 (0.02)	−0.12^***^ (0.01)
Physical self-concept (PSC)				0.24^***^ (0.04)		0.39^***^ (0.05)	0.38^***^ (0.04)	0.24^***^ (0.04)
Intrinsic motivation (INTR)	0.33^***^ (0.05)			0.08^*^ (0.04)				0.08^*^ (0.04)
Extrinsic motivation (EXTR)	0.32^***^ (0.04)			−0.02 (0.04)				−0.03 (0.04)
**Standardized indirect effects**								
INTR→ PSC →				0.08^***^ (0.02)				
EXTR→ PSC →				0.08^***^ (0.02)				
PSC→ INTR→								0.03^*^ (0.01)
PSC→ EXTR→								−0.01 (0.01)
*R^2^*				0.23				0.23

*PP, physical performance*;

* *p < 0.05*,

** *p < 0.01*,

*** *p < 0.001. R^2^, explained variance*.

In Model 1 assuming physical self-concept as a mediator of the relations between both types of motivation and physical performance, physical self-concept proved to significantly mediate the relations of both types of motivation to physical performance. In contrast, in Model 2 assuming both types of motivation as mediators of the relation between physical self-concept and physical performance, only intrinsic motivation proved to significantly mediate the relation between physical self-concept and physical performance. The direct relations were similar in both mediation models and to those of the SEM model without indirect relations: Both physical self-concept and intrinsic motivation were significantly positively linked to physical performance, while extrinsic motivation was slightly negatively but not significantly linked to physical performance. Girls and children with a higher BMI showed a lower total performance score than boys and children with a lower BMI. However, only the regression path for girls' lower physical self-concept remained substantial in Model 2, including the mediation of both types of motivation. In contrast, the regression paths for the children's BMI remained significant in both mediation models. Furthermore, in both mediation models, physical self-concept was significantly positively related to both types of motivation. The amount of explained variance was.23 in both mediation models, and the fit of both mediation models was also good (see Table 2).

## Discussion

Based on a very neglected sample of 1,082 children aged 7–8 years, the present study sought to examine the relations between physical self-concept, intrinsic and extrinsic motivation as well as physical performance. Beyond the large sample size, the incremental contribution of this study was that two mediation models were tested simultaneously: (a) a mediation model assuming physical self-concept as a mediator of the relations between both types of motivation and physical performance and (b) a mediation model assuming both types of motivation as mediators of the relations between physical self-concept and physical performance.

In support of previous research (e.g., Marsh et al., 2006a,b; Trautwein et al., 2008; Standage et al., 2012; Wang et al., 2016; Garn et al., 2019; Vasconcellos et al., 2020), physical self-concept was significantly positively related to intrinsic motivation and physical performance. However, not fully in line with hypothesis 1 and previous results (e.g., Vallerand, 2007; Boiché et al., 2008; Gillet et al., 2010), physical self-concept was also significantly positively related to extrinsic motivation. In contrast and consistent with Hypothesis 2, results of SEM revealed that physical self-concept and intrinsic motivation were significantly positively linked to physical performance, while extrinsic motivation was slightly but not significantly negatively linked to physical performance. The negative regression path of extrinsic motivation may result from the typically higher levels of intrinsic motivation of younger children, as also evidenced in the descriptive analysis of this study. Furthermore, in accordance with Hypothesis 3, results also provided support for both mediation models: physical self-concept proved to significantly mediate the relations of both types of motivation to physical performance. In contrast, only intrinsic motivation proved to significantly mediate the relation between physical self-concept and physical performance, while extrinsic motivation did not play a significant mediating role in predicting physical performance. A possible reason for these findings is that younger children are typically more motivated in physical activities than older children (e.g., Xiang et al., 2004; Gao et al., 2008), as also indicated by the increasing decline of participation in physical activities during adolescence [see the review by Eime et al. (2016)]. To help younger children develop a healthier lifestyle, which is one of the central objectives of the Fulda Movement Check project, children's physical self-concept and intrinsic motivation should be supported in early childhood, when children's physical self-concept and motivation mainly develop (Harter, 2012). In contrast, interventions aiming at improving children's physical performance only by means of an increased level of physical activity without enhancing children's physical self-concept may have less or no effects on their physical performance. However, results of this study also yielded a slightly negative regression path of extrinsic motivation on physical performance, indicating that extrinsic motivation is rather detrimental for children's physical performance. In addition, girls and children with a higher BMI had a significantly lower physical self-concept and a lower total performance score than boys and children with a lower BMI, as also earlier shown in previous studies (e.g., Deaner et al., 2016; Ferreira et al., 2019; Queiroz et al., 2020). By implication, girls and children with a higher BMI are at risk and should be supported in physical activities by, for instance, specific physical tests related to their individual motor skills to sustainably motivate them to more physical activities and a healthier lifestyle.

### Limitations and Future Directions

Although results of this study provide great insight into the interplay of younger children's physical self-concept, motivation, and physical performance, some limitations must be warranted. The first limitation concerns the cross-sectional data which do not provide solid evidence for causality. Second, only a small number of variables were taken into consideration in the regression analyses under study. Further studies should therefore measure additional variables to increase the explained variance in children's physical performance and to provide a more comprehensive picture of the underlying processes that account for the varying levels of children's physical performance. In particular, there is a deficit of psychosocial variables, such as the level of physical activity, and family or home environment factors, such as the relationships with parents and peers, which also influence children's physical performance [Barnett et al., 2019; see the meta-analysis by Barnett et al. (2016)]. Third, despite the large sample size, results of this study are only representative for children aged 7–8 years. For this reason, no implications can be drawn for older children, and further studies with more heterogenous age groups must show how the relations change during childhood and adolescence. Keeping especially in mind that achievement mainly influences self-concept in early childhood and that self-concept is more likely to reciprocally relate to achievement at the end of elementary school (Guay et al., 2003; Chen et al., 2013), longitudinal studies should test the relations at different developmental stages. Furthermore, since the tests of the Fulda Movement Check project took place outside of school in different sports halls, no data of the classes, in which the children were drawn, were available. As a consequence, it was not possible to perform multilevel analyses or to take the hierarchical data (i.e., students in classes) into account, which increases the possibility of inflated standard errors.

### Implications and Conclusion

Despite the limitations mentioned, results of this study are extensible to a very specific segment of the population, favoring the individualization of intervention strategies in the field of physical education and sports, and provide some important implications for sport coaches and physical teachers. A first implication targets the mediation models under investigation. While physical self-concept proved to significantly mediate the relations of both types of motivation to physical performance, only intrinsic motivation proved to significantly mediate the relation between physical self-concept and physical performance. This is a novel finding which has not been stated in previous research so far. An important implication of this finding is that children who are intrinsically motivated in physical activities only perform better in motor skill tests when their physical self-concept is high. Furthermore, the significant mediating effect of intrinsic motivation suggests that children who feel competent in physical activities perform better when they are intrinsically motivated, that is performing physical activities for their inherent pleasure without any external pressure. By implication, sport coaches or physical teachers should avoid extrinsic contingencies such as rewards or good grades. Finally, of great concern is the lower physical self-concept and poorer physical performance of girls and children with a higher BMI. Appropriate interventions are therefore needed to support the physical self-concept and physical skills of girls and children with a higher BMI. For instance, devising appropriate instructions of arousing children's physical self-concept and giving continuous feedback by using individual reference norms are effective strategies to promote children's physical self-concepts and improve their physical performance (Schmidt et al., 2013).

In conclusion, results of this investigation replicate and expand previous literature by showing that it is predominantly children's physical self-concept and intrinsic motivation that determine their physical performance and play a mediating role in predicting their physical performance. Interventions targeted at improving younger children's physical performance should thus establish conditions to promote children's physical self-concept and intrinsic motivation by means of adapting the level of motor skill tests to children's individual physical abilities, providing positive feedback to their physical performance, and enhancing their individual progress (Deci and Ryan, 2002). This may be of particular importance for girls and children with a higher BMI who showed a much lower physical self-concept and poorer physical performance in this study and many other studies (e.g., Morano et al., 2011; Zsakai et al., 2017; Ferreira et al., 2019; Queiroz et al., 2020).

## Data Availability Statement

The raw data supporting the conclusions of this article will be made available by the authors, without undue reservation.

## Ethics Statement

Ethical review and approval was not required for the study on human participants in accordance with the local legislation and institutional requirements. Written informed consent to participate in this study was provided by the participants' legal guardian/next of kin.

## Author Contributions

AL developed the measures of self-concept and motivation, cleaned the data, performed the data analysis, and wrote the initial draft of the manuscript. PK recruited the sample, developed the study concept, collected the data, and reviewed the manuscript. MD and AH developed the study concept and reviewed the manuscript. All authors read and approved the submitted manuscript.

## Conflict of Interest

The authors declare that the research was conducted in the absence of any commercial or financial relationships that could be construed as a potential conflict of interest.

## Footnotes

^1^Lohbeck, A., Istvan, T.-K., and Morin, A. (2021). Latent motivation profiles of elementary school children across math and German domains: A self-determination theory approach.

## References

[B1] Arens A. K. Marsh H. W. Craven R. G. Yeung A. S. Randhawa E. Hasselhorn M. (2016). Math self-concept in preschool children: structure, achievement relations, and generalizability across gender. Early Childhood Res. Q. 36, 391–403. 10.1016/j.ecresq.2015.12.024

[B2] Barnett L. M. Hnatiuk J. A. Salmon J. Hesketh K. D. (2019). Modifiable factors which predict children's gross motor competence: a prospective cohort study. Int. J. Beh. Nutr. Phys. Act. 16, 1–11. 10.1186/s12966-019-0888-0 31829267 PMC6907285

[B3] Barnett L. M. Lai S. K. Veldman S. L. C. Hardy L. L. Cliff D. P. Morgan P. J.. (2016). Correlates of gross motor competence in children and adolescents: a systematic review and meta-analysis. Sports Med. 46, 1663–1688. 10.1007/s40279-016-0495-z 26894274 PMC5055571

[B4] Bodzsar E. B. Zsakai A. (2014). Recent trends in childhood obesity and overweight in the transition countries of Eastern and Central Europe. Ann. Human Biol. 41, 263–270. 10.3109/03014460.2013.856473 24702625

[B5] Boes K. Schlenker L. (2016). Der Deutsche Motorik-Test 6-18 [The German Motor Skills Test]. Hamburg: Feldhaus.

[B6] Boiché J. C. S. Sarrazin P. G. Grouzet F. M. E. Pelletier L. G. Chanal J. P. (2008). Students' motivational profiles and achievement outcomes in physical education: a self-determination perspective. J. Educ. Psychol. 100, 688–701. 10.1037/0022-0663.100.3.688

[B7] Brown K. A. Patel D. R. Darmawan D. (2017). Participation in sports in relation to adolescent growth and development. Transl. Pediatrics 6, 150–159. 10.21037/tp.2017.04.03 28795005 PMC5532200

[B8] Cerasoli C. P. Nicklin J. M. Ford M. T. (2014). Intrinsic motivation and extrinsic incentives jointly predict performance: a 40-year meta-analysis. Psychol. Bull. 140, 980–1008. 10.1037/a0035661 24491020

[B9] Chen S.-K. Yeh Y.-C. Hwang F.-M. Lin S. S. J. (2013). The relationship between academic self-concept and achievement: a multicohort-multioccasion study. Learn. Individ. Dif. 23, 172–178. 10.1016/j.lindif.2012.07.021

[B10] Craggs C. Corder K. van Sluijs E. M. F. Griffin S. J. (2011). Determinants of change in physical activity in children and adolescents: a systematic review. Am. J. Prev. Med. 40, 645–658. 10.1016/j.amepre.2011.02.025 21565658 PMC3100507

[B11] Cumming S. P. Standage M. Loney T. Gammon C. Neville H. Sherar L. B.. (2011). The mediating role of physical self-concept on relations between biological maturity status and physical activity in adolescent females. J. Adolesc. 34, 465–473. 10.1016/j.adolescence.2010.06.006 20655102

[B12] Deaner R. O. Balish S. M. Lombardo M. P. (2016). Sex differences in sports interest and motivation: An evolutionary perspective. Evolution. Beh. Sci. 10, 73–97. 10.1037/ebs0000049

[B13] Deci E. L. Ryan R. M. (2002). Handbook of Self-Determination Research. Rochester, NY: University of Rochester Press.

[B14] Eccles J. S. Harold R. D. (1991). Gender differences in sport participation: applying the Eccles' expectancy model. J. Appl. Sports Psychol. 3, 7–35. 10.1080/10413209108406432

[B15] Eccles J. S. Wigfield A. (2020). From expectancy-value theory to situated expectancy-value theory: a developmental, social cognitive, and sociocultural perspective on motivation. Contemp. Educ. Psychol. 61:101859. 10.1016/j.cedpsych.2020.101859

[B16] Eime R. M. Harvey J. T. Charity M. J. Payne W. R. (2016). Population levels of sport participation: implications for sport policy. BMC Public Health 16, 1–8. 10.1186/s12889-016-3463-5 27506922 PMC4977647

[B17] Eime R. M. Young J. A. Harvey J. T. Charity M. J. Payne W. R. (2013). A systematic review of the psychological and social benefits of participation in sport for children and adolescents: informing development of a conceptual model of health through sport. Int. J. Beh. Nutr. Phys. Act. 10, 1–21. 10.1186/1479-5868-10-98 23945179 PMC3751802

[B18] Fernández-Bustos J. G. Infantes-Paniagua Á. Cuevas R. Contreras O. R. (2019). effect of physical activity on self-concept: theoretical model on the mediation of body image and physical self-concept in adolescents. Front. Psychol. 10:1537. 10.3389/fpsyg.2019.01537 31354570 PMC6635469

[B19] Ferreira L. Vieira J. L. L. Silva P. N. D. Chaves R. N. D. Fernandes R. A. Cheuczuk F.. (2019). The role of sport participation and body mass index in predicting motor competence of school-age children. J. Phys. Educ. 30:e3024. 10.4025/jphyseduc.v30i1.3024

[B20] Freund P. A. Lohbeck A. (2020). Modeling self-determination theory motivation data by using unfolding IRT. Eur. J. Psychol. Ass. 10.1027/1015-5759/a000629. [Epub ahead of print].

[B21] Gao Z. Lee A. M. Harrison L. (2008). Understanding students' motivation in sport and physical education: From the expectancy-value model and self-efficacy theory perspectives. Quest 60, 236–254. 10.1080/00336297.2008.10483579

[B22] Garn A. C. Moore E. W. Centeio E. E. Kulik N. Somers C. McCaughtry N. (2019). Reciprocal effects model of children's physical activity, physical self-concept, and enjoyment. Psychol. Sport Exerc. 45:101568. 10.1016/j.psychsport.2019.101568 27385738

[B23] Gillet N. Vallerand R. J. Amoura S. Baldes B. (2010). Influence of coaches' autonomy support on athletes' motivation and sport performance: a test of the hierarchical model of intrinsic and extrinsic motivation. Psychol. Sport Exerc. 11, 155–161. 10.1016/j.psychsport.2009.10.004

[B24] Guay F. Marsh H. W. Boivin M. (2003). Academic self-concept and academic achievement: developmental perspectives on their causal ordering. J. Educ. Psychol. 95, 124–136. 10.1037/0022-0663.95.1.124

[B25] Guay F. Ratelle C. F. Chanal J. (2008). Optimal learning in optimal contexts: the role of self-determination in education. Can. Psychol. 49, 233–240. 10.1037/a0012758

[B26] Harter S. (2012). The Construction of Self: Developmental and Sociocultural Foundations. New York, NY: Guilford Press.

[B27] Hawes M. R. Martin A. D. (2001). Human body composition, in Kinanthropometry and Exercise Physiology Laboratory Manual: Tests, Procedures and Data, Vol. I, eds Norton K. Eston R. (London: Routledge).

[B28] Hohmann A. Siener M. He R. (2018). Prognostic validity of talent orientation in soccer. Germ. J. Exerc. Sport Res. 48, 478–488. 10.1007/s12662-018-0549-5

[B29] Hohmann A. Zapp M. Hohmann L. Scheuring L. (2016). Fuldaer Bewegungscheck. Vorläufiger Ergebnisbericht 2010–2015. [Fulda Movement Check. Preliminary report of results]. Available online at: https://www.spowi1.uni-bayreuth.de/de/forschung/Projekte/index.html

[B30] Hu L. Bentler P. M. (1999). Cutoff criteria for fit indexes in covariance structure analysis: Conventional criteria versus new alternatives. Struct. Equ. Mod. 6, 1–55. 10.1080/10705519909540118

[B31] Jacobs J. E. Lanza S. Osgood D. W. Eccles J. S. Wigfield A. (2002). Changes in children's self-competence and values: gender and domain differences across grades one through twelve. Child Dev. 73, 509–527. 10.1111/1467-8624.00421 11949906

[B32] Klein M. Froehlich M. Emrich E. (2012). Zur Testgenauigkeit ausgewählter Items des Deutschen Motorik-Tests DMT 6-18 [On the precision of selected items of the German Motor Test 6-18]. Leipziger Sportwissenschaftliche Beiträge 53, 23–45.

[B33] Lakes K. D. Neville R. D. Abdullah M. Donnelly J. (2020). Psychological determinants of physical activity and development in early childhood among children with developmental delays: the role of parent beliefs regarding the benefits of physical activity. Front. Sports Act. Liv. 2:104. 10.3389/fspor.2020.00104 33345093 PMC7739724

[B34] Marsh H. W. (1990). The Self-Description Questionnaire I: SDQ I manual. Macarthur, NSW: University of Western Sydney. 10.1037/t01843-000

[B35] Marsh H. W. (2007). Self-Concept Theory, Measurement and Research Into Practice: The Role of Self-Concept in Educational Psychology. Leicester: British Psychological Society.

[B36] Marsh H. W. Chanal J. P. Sarrazin P. G. (2006a). Self-belief does make a difference: a reciprocal effects model of the causal ordering of physical self-concept and gymnastics performance. J. Sports Sci. 24, 101–111. 10.1080/02640410500130920 16368618

[B37] Marsh H. W. Craven R. Debus R. (1998). Structure, stability, and development of young children's self-concepts: a multicohort-multioccasion study. Child Dev. 69, 1030–1053. 10.1111/j.1467-8624.1998.tb06159.x 9768485

[B38] Marsh H. W. Craven R. G. Debus R. (1991). Self-concepts of young children aged 5 to 8: their measurement and multidimensional structure. J. Educ. Psychol. 83, 377–392. 10.1037/0022-0663.83.3.377

[B39] Marsh H. W. Ellis L. A. Craven R. G. (2002). How do preschool children feel about themselves? Unraveling measurement and multidimensional self-concept structure. Dev. Psychol. 38, 376–393. 10.1037/0012-1649.38.3.376 12005381

[B40] Marsh H. W. Gerlach E. Trautwein U. Lüdtke O. Brettschneider W. D. (2007a). Longitudinal study of preadolescent sport self-concept and performance: reciprocal effects and causal ordering. Child Dev. 78, 1640–1656. 10.1111/j.1467-8624.2007.01094.x 17988312

[B41] Marsh H. W. Hau K. T. Sung R. Y. Yu C. W. (2007b). Childhood obesity, gender, actual-ideal body image discrepancies, and physical self-concept in Hong Kong children: cultural differences in the value of moderation. Dev. Psychol. 43, 647–662. 10.1037/0012-1649.43.3.647 17484577

[B42] Marsh H. W. Papaioannou A. Theodorakis Y. (2006b). Causal ordering of physical self-concept and exercise behavior: reciprocal effects model and the influence of physical education teachers. Health Psychol. 25, 316–328. 10.1037/0278-6133.25.3.316 16719603

[B43] Marsh H. W. Redmayne R. S. (1994). A multidimensional physical self-concept and its relations to multiple components of physical fitness. J. Sport Exerc. Psychol. 16, 43–55. 10.1123/jsep.16.1.43

[B44] Marsh H. W. Trautwein U. Lüdtke O. Köller O. Baumert J. (2005). Academic self-concept, interest, grades, and standardized test scores: reciprocal effects models of causal ordering. Child Dev. 76, 397–416. 10.1111/j.1467-8624.2005.00853.x 15784090

[B45] McDonough M. H. Crocker P. R. (2007). Testing self-determined motivation as a mediator of the relationship between psychological needs and affective and behavioral outcomes. J. Sport Exerc. Psychol. 29, 645–663. 10.1123/jsep.29.5.645 18089897

[B46] Mendo-Lázaro S. Polo-del-Río M. I. Amado-Alonso D. Iglesias-Gallego D. León-del-Barco B. (2017). Self-concept in childhood: the role of body image and sport practice. Front. Psychol. 8:853. 10.3389/fpsyg.2017.00853 28596750 PMC5443145

[B47] Morano M. Colella D. Robazza C. Bortoli L. Capranica L. (2011). Physical self-perception and motor performance in normal-weight, overweight and obese children. Scand. J. Med. Sci. Sports 21, 465–473. 10.1111/j.1600-0838.2009.01068.x 20136752

[B48] Muthén L. K. Muthén B. O. (1998–2018). Mplus User's Guide (8th ed.). Los Angeles, CA: Muthén and Muthén.

[B49] Nicholls J. G. (1979). Development of perception of own attainment and causal attributions for success and failure in reading. J. Educ. Psychol. 71, 94–99. 10.1037/0022-0663.71.1.94 438417

[B50] Ntoumanis N. (2001). A self-determination approach to the understanding of motivation in physical education. Br. J. Educ. Psychol. 71, 225–242. 10.1348/000709901158497 11449934

[B51] Ntoumanis N. Standage M. (2009). Motivation in physical education classes: a self-determination theory perspective. Theory Res. Educ. 7, 194–202. 10.1177/1477878509104324

[B52] Onetti-Onetti W. Chinchilla-Minguet J. L. Martins F. M. L. Castillo-Rodriguez A. (2019). Self-concept and physical activity: differences between high school and University students in Spain and Portugal. Front. Psychol. 10:1333. 10.3389/fpsyg.2019.01333 31281276 PMC6596367

[B53] Paeratakul S. White M. A. Williamson D. A. Ryan D. H. Bray G. A. (2002). Sex, race/ethnicity, socioeconomic status, and BMI in relation to self-perception of overweight. Obes. Res. 10, 345–350. 10.1038/oby.2002.48 12006633

[B54] Queiroz D. D. R. Aguilar J. A. Martins Guimarães T. G. Hardman C. M. Lima R. A. Duncan M. J.. (2020). Association between body mass index, physical activity and motor competence in children: moderation analysis by different environmental contexts. Annals of Human Biol. 47, 417–424. 10.1080/03014460.2020.1779815 32613892

[B55] Richter S. Tietjens M. Ziereis S. Querfurth S. Jansen P. (2016). Yoga training in junior primary school-aged children has an impact on physical self-perceptions and problem-related behavior. Front. Psychol. 7:203. 10.3389/fpsyg.2016.00203 26941676 PMC4763067

[B56] Rodriguez-Ayllon M. Cadenas-Sánchez C. Estévez-López F. Muñoz N. E. Mora-Gonzalez J. Migueles J. H.. (2019). Role of physical activity and sedentary behavior in the mental health of preschoolers, children and adolescents: a systematic review and meta-analysis. Sports Med. 49, 1383–1410. 10.1007/s40279-019-01099-5 30993594

[B57] Ryan R. M. Connell J. P. (1989). Perceived locus of causality and internalization: examining reasons for acting in two domains. J. Pers. Soc. Psychol. 57, 749–761. 10.1037/0022-3514.57.5.749 2810024

[B58] Schmidt M. Valkanover S. Roebers C. Conzelmann A. (2013). Promoting a functional physical self-concept in physical education: evaluation of a 10-week intervention. Eur. Phys. Educ. Rev. 19, 232–255. 10.1177/1356336X13486057

[B59] Schmutz E. A. Leeger-Aschmann C. S. Kakebeeke T. H. Zysset A. E. Messerli-Bürgy N. Stülb K.. (2020). Motor competence and physical activity in early childhood: stability and relationship. Front. Public Health 8:39. 10.3389/fpubh.2020.00039 32154207 PMC7047434

[B60] Shavelson R. J. Hubner J. Stanton G. C. (1976). Validation of construct interpretation. Rev. Educ. Res. 46, 407–441. 10.3102/00346543046003407

[B61] Sproule J. Wang C. K. J. Morgan K. McNeill M. McMorris T. (2007). Effects of motivational climate in Singaporean physical education lessons on intrinsic motivation and physical activity intention. Pers. Individ. Dif. 43, 1037–1049. 10.1016/j.paid.2007.02.017

[B62] Standage M. Duda J. L. Ntoumanis N. (2003). A model of contextual motivation in physical education: Using constructs from self-determination and achievement goal theories to predict physical activity intentions. J. Educ. Psychol. 95, 97–110. 10.1037/0022-0663.95.1.97

[B63] Standage M. Gillison F. B. Ntoumanis N. Treasure D. C. (2012). Predicting students' physical activity and health-related well-being: a prospective cross-domain investigation of motivation across school physical education and exercise settings. J. Sport Exerc. Psychol. 34, 37–60. 10.1123/jsep.34.1.37 22356882

[B64] Standage M. Sebire S. J. Loney T. (2008). Does exercise motivation predict engagement in objectively assessed bouts of moderate-intensity exercise? A self-determination theory perspective. J. Sport Exerc. Psychol. 30, 337–352. 10.1123/jsep.30.4.337 18723896

[B65] Stewart A. Marfell-Jones M. Olds T. de Ridder H. (2011). International Standards for Anthropometric Assessment. Wellington: ISAK.

[B66] Stipek D. J. MacIver D. M. (1989). Developmental changes in children's assessment of intellectual competence. Child Dev. 60, 521–538. 10.2307/1130719

[B67] Thøgersen-Ntoumani C. Ntoumanis N. (2006). The role of self-determined motivation in the understanding of exercise-related behaviours, cognitions and physical self-evaluations. J. Sports Sci. 24, 393–404. 10.1080/02640410500131670 16492603

[B68] Trautwein U. Gerlach E. Lüdtke O. (2008). Athletic classmates, physical self-concept, and free-time physical activity: a longitudinal study of frame of reference effects. J. Educ. Psychol. 100, 988–1001. 10.1037/0022-0663.100.4.988

[B69] Trost S. G. Kerr L. M. Ward D. S. Pate R. R. (2001). Physical activity and determinants of physical activity in obese and non-obese children. Int. J. Obes. 25, 822–829. 10.1038/sj.ijo.0801621 11439296

[B70] Utesch T. Dreiskämper D. Strauss B. Naul R. (2018). The development of the physical fitness construct across childhood. Scand. J. Med. Sci. Sports 28, 212–219. 10.1111/sms.12889 28376240

[B71] Vallerand R. J. (2007). Intrinsic and extrinsic motivation in sport and physical activity. A review and a look at the future, in Handbook of Sport Psychol, eds. Tenenbaum G. Eklund R. C. (New York, NY: John Wiley and Sons), 59–83. 10.1002/9781118270011.ch3

[B72] Vasconcellos D. Parker P. D. Hilland T. Cinelli R. Owen K. B. Kapsal N.. (2020). Self-determination theory applied to physical education: a systematic review and meta-analysis. J. Educ. Psychol. 112, 1444–1469. 10.1037/edu000042031649571

[B73] Wang J. C. Morin A. J. Liu W. C. Chian L. K. (2016). Predicting physical activity intention and behaviour using achievement goal theory: a person-centered analysis. Psychol. Sport Exerc. 23, 13–20. 10.1016/j.psychsport.2015.10.004

[B74] Wigfield A. (1994). Expectancy-value theory of achievement motivation: a developmental perspective. Educ. Psychol. Rev. 6, 49–78. 10.1007/BF02209024

[B75] World Health Organization (2016). Childhood Overweight and Obesity. Geneva: World Health Organization.

[B76] Xiang P. McBride R. Guan J. (2004). Children's motivation in elementary physical education: a longitudinal study. Res. Q. Exerc. Sport 75, 71–80. 10.1080/02701367.2004.10609135 15532363

[B77] Zsakai A. Karkus Z. Utczas K. Bodzsar E. B. (2017). Body structure and physical self-concept in early adolescence. J. Early Adolesc. 37, 316–338. 10.1177/027243161560275730583277

